# Cancer vaccines: what’s next?

**DOI:** 10.18632/oncotarget.27006

**Published:** 2019-06-18

**Authors:** Stéphane Depil, Paola Bonaventura, Vincent Alcazer

**Affiliations:** Centre Léon Bérard, Lyon, France; Inserm U1052/CNRS 5286, Centre de Recherche en Cancérologie de Lyon, Lyon, France

**Keywords:** personalised cancer vaccine, neoepitope, immunotherapy

**Figure 1:**
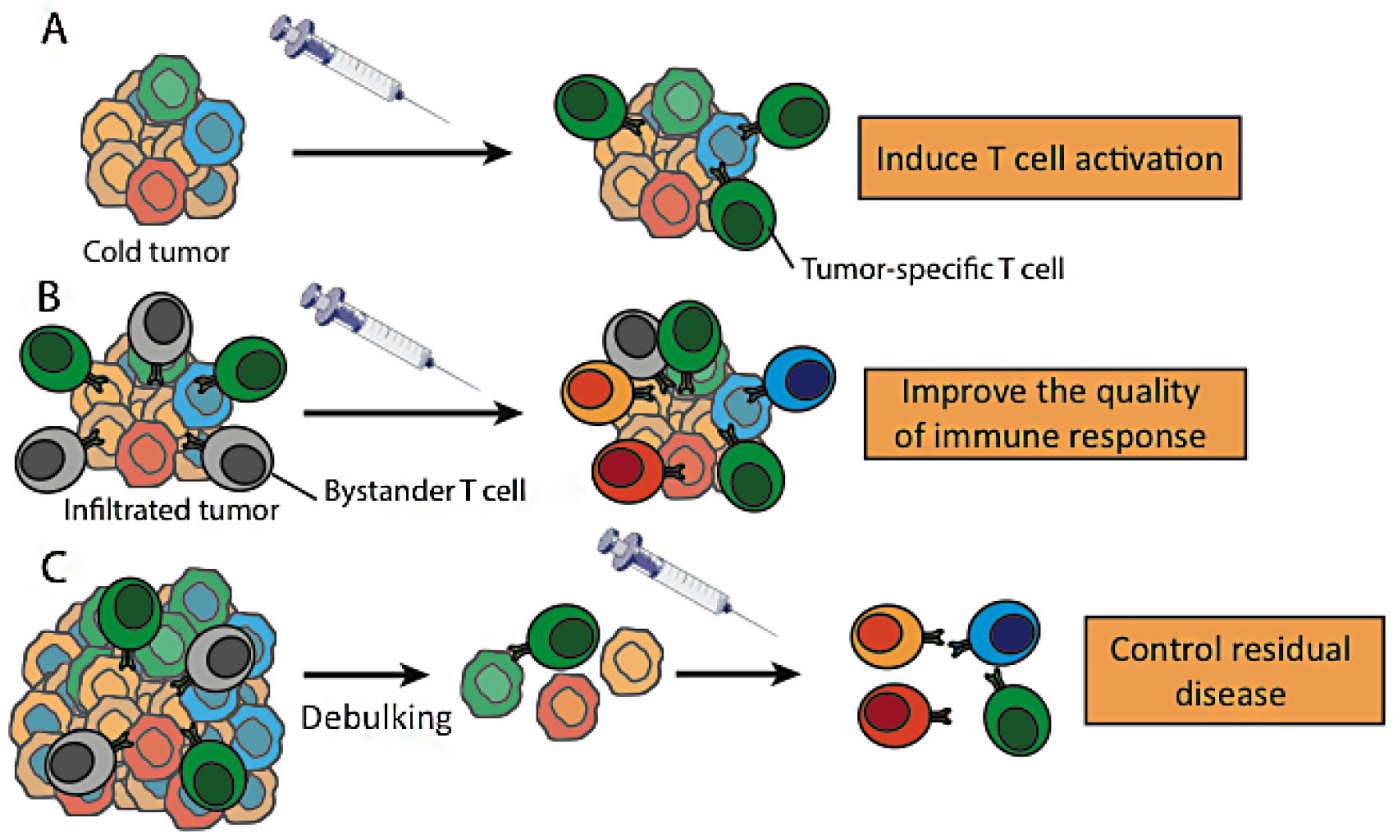
**Potential effects of cancer vaccination in immunotherapy.** Therapeutic cancer vaccines, by inducing tumor-specific T cells, may turn non-inflamed/cold tumors into inflamed/hot tumors **(A)** or improve the quality of the T cell infiltrate in inflamed tumors **(B)**. Cancer vaccination may be also used to control or eliminate a residual disease persisting after conventional antitumor therapies **(C)**.

Disappointing results have been observed with the first generation therapeutic cancer vaccines. However, the renaissance of immunotherapy with the checkpoint inhibitors and the demonstration, in preclinical models, that mutation-associated neoantigens can induce efficient antitumor T cell responses, have led to a renewed interest in cancer vaccination. Given its potential to both induce a specific T cell response from naïve T cells and to amplify a pre-existing antitumor immune response, personalized vaccination based on tumor mutations-derived neoepitopes is a promising approach in the field of immunotherapy. Cancer vaccination may turn cold tumors into hot ones by inducing a T cell response inside the tumor, a prerequisite for the efficacy of PD-1/-L1 antagonists [1]. In tumors already infiltrated by T cells, vaccination may improve the quality of the immune response by increasing the number of tumor-reactive T cells comparatively with bystander T cells. Finally, there is also a strong rationale to develop cancer vaccines in the adjuvant setting, to allow a long-term control of the residual disease by the immune system (Figure 1).

The first results obtained in the clinic with neoepitopes-based vaccines have been recently published [2–5]. The authors used mRNA-based vaccine in melanoma or synthetic long peptides in melanoma and glioblastoma. These studies provide a proof of concept that T cell responses can be induced against the neoepitopes incorporated in the vaccines, which may be associated with a clinical benefit, at least in melanoma studies. However, further studies are still needed to confirm the clinical efficacy of the approach. Furthermore, it is surprising to observe that, in all these different studies, the vaccine seemed to induce preferentially CD4^+^ T cells rather than the expected CD8^+^ T cell responses.

We have recently discussed the main challenges associated with the development of neoepitopes-based vaccines [6]. We will consider here five major questions that must be answered for the success of future clinical developments:

- What is the optimal method to select neoepitopes and product personalized vaccines? Classical prediction tools are probably not sufficient to select the best candidates, especially for MHC class II epitopes. Furthermore, a significant proportion of the predicted MHC class I neoepitopes may not be presented by the tumor cells. Several groups are working to improve prediction algorithms by integrating new parameters, such as mass spectrometry datasets combined with machine learning approaches, to develop better predictive models [7]. Optimizing this step is also a key point to decrease the overall time of the production process, which is another major challenge. From the patient’s sample collection to the release of the personalized vaccine, the entire process currently takes approximately 3 to 4 months, requiring additional patient treatments before the availability of the vaccine. An optimized production process together with improved neoepitopes selection may reduce this time to less than 6 weeks.
- How to deal with clonal heterogeneity? Among the different immune escape mechanisms, the selection of tumor clones that do not express the vaccine epitopes is a major concern. Even if epitope spreading mechanisms can extend the immune response to other tumor epitopes after vaccination, one can propose to prioritize clonal neoepitopes, or a mixture of neoepitopes representative of the main subclones, in order to limit such immune selection.
- What is the best formulation to induce strong CD8^+^ T cell responses? Even if Th1 CD4^+^ T cells play a major role in antitumor immunity, it is probably necessary to induce a sufficient number of cytotoxic CD8^+^ T cells to provide an effective antitumor response. In this context, viral vectors may represent an interesting vaccine format and comparisons should be made between synthetic long peptides, mRNA, viral vectors and attenuated bacterial vectors.
- What is the best way to efficiently combine cancer vaccine with immune checkpoint inhibitors? An optimal sequencing may be critical. Whereas anti-CTLA4 may be used concurrently with vaccines to enhance T cell priming, this might be slightly different for PD-1/-L1 antagonists. There are arguments to consider that an early concomitant use of a PD-1/-L1 antagonist may be useful to increase T cell activation at the initial phase, but also some preclinical data suggesting that it should be administered later, after the priming phase, since PD-L1/PD-1 inhibition may interfere with the quality of the T cell response [8]. Finally, other checkpoint modulators or drugs capable of inhibiting suppressive cells may be envisioned. For instance, some trials have used prior cyclophosphamide administration to reduce the number of regulatory T cells.
- What is the optimal timing and clinical positioning of cancer vaccines? As classically done in cancer drug development, the first studies were performed in advanced or metastatic diseases. However, a high tumor burden, which is associated with a higher clonal heterogeneity and immunosuppression, may impair vaccine efficacy in this context. An earlier administration may reduce the risk of facing all the different mechanisms of immunoediting and immune resistance developed during tumor progression, including loss of β2-microglobulin, loss of heterozygosity of HLA alleles presenting neoepitopes and depletion of the expressed neoantigens [9]. There is therefore a strong rationale to use cancer vaccination in consolidation after debulking therapies, or in the adjuvant setting at an early stage of the disease, which is currently being investigated by more and more studies.

Considering all the efforts provided by academic and pharmaceutical groups worldwide, we could be optimistic that the main challenges associated with the development of these next generation cancer vaccines will be overcome. It should be noted that we mainly focused here on neoepitopes derived from single nucleotide variants. Other families of tumor antigens are of great interest and should be evaluated in the near future, such as neoepitopes derived from gene fusions or small insertions and deletions, or other types of epitopes derived from cancer-associated epigenetic, transcriptional, translational or post-translational aberrations [10]. We are still at the beginning of the story.
